# CRISPR-Cas-Docker: web-based in silico docking and machine learning-based classification of crRNAs with Cas proteins

**DOI:** 10.1186/s12859-023-05296-y

**Published:** 2023-04-25

**Authors:** Ho-min Park, Jongbum Won, Yunseol Park, Esla Timothy Anzaku, Joris Vankerschaver, Arnout Van Messem, Wesley De Neve, Hyunjin Shim

**Affiliations:** 1 Center for Biosystems and Biotech Data Science, Ghent University Global Campus, Incheon, 21985 South Korea; 2 Department of Electronics and Information Systems, Ghent University, 9000 Ghent, Belgium; 3 Department of Applied Mathematics, Computer Science and Statistics, Ghent University, 9000 Ghent, Belgium; 4 Department of Mathematics, University of Liège, 4000 Liège, Belgium

**Keywords:** CRISPR-Cas systems, In silico docking, Protein tertiary structure, RNA tertiary structure, RNA secondary structure, Machine learning-based classification, CRISPR direct repeat

## Abstract

**Background:**

CRISPR-Cas-Docker is a web server for in silico docking experiments with CRISPR RNAs (crRNAs) and Cas proteins. This web server aims at providing experimentalists with the optimal crRNA-Cas pair predicted computationally when prokaryotic genomes have multiple CRISPR arrays and Cas systems, as frequently observed in metagenomic data.

**Results:**

CRISPR-Cas-Docker provides two methods to predict the optimal Cas protein given a particular crRNA sequence: a structure-based method (in silico docking) and a sequence-based method (machine learning classification). For the structure-based method, users can either provide experimentally determined 3D structures of these macromolecules or use an integrated pipeline to generate 3D-predicted structures for in silico docking experiments.

**Conclusion:**

CRISPR-Cas-Docker addresses the need of the CRISPR-Cas community to predict RNA–protein interactions in silico by optimizing multiple stages of computation and evaluation, specifically for CRISPR-Cas systems. CRISPR-Cas-Docker is available at www.crisprcasdocker.org as a web server, and at https://github.com/hshimlab/CRISPR-Cas-Docker as an open-source tool.

**Supplementary Information:**

The online version contains supplementary material available at 10.1186/s12859-023-05296-y.

## Background

CRISPR-Cas is a prokaryotic adaptive immune system [1, 2] that consists of two genetic components: (1) CRISPR arrays with CRISPR RNAs (crRNAs) encompassing short palindromic repeats and unique spacers from previous infections and (2) CRISPR-associated systems (Cas) which form a complex of proteins to cleave invading foreign genetic elements. CRISPR-Cas systems have been repurposed as genome-editing tools [3, 4] and antimicrobials [5, 6], with this biotechnological potential driving the scientific community to discover novel types of CRISPR-Cas systems [7–9].

CRISPR arrays are assumed to be associated with Cas systems when they are co-located in prokaryotic genomes (usually within ± 10,000 base pairs). However, metagenomic data from diverse environments have revealed that prokaryotic genomes often have multiple CRISPR arrays and Cas systems. Such complexity in genomic architecture can lead to suboptimal RNA–protein interactions between the crRNA-Cas protein complex in CRISPR-Cas-based genomic tools [10]. In a previous study, we predicted crRNAs that bind optimally to a particular Cas protein through in silico docking experiments, suggesting that such in silico experiments can be adopted as a preliminary approach to design stable CRISPR-based antimicrobials using the newly discovered Cas13 proteins [11].

Here, we present a web application named CRISPR-Cas-Docker that offers an optimized and integrated pipeline to conduct in silico docking experiments between a crRNA and a Cas protein (Additional file 1: Fig. S1). By leveraging our expertise with RNA structure prediction, AlphaFold-based protein structure prediction, and in silico macromolecular docking, we aim at providing experimentalists with a practical and user-friendly bioinformatics tool that can suggest the most optimal crRNA-Cas protein pairs to be tested in vitro.

## Implementation and results

### Predicting the 3D structures of crRNAs and Cas proteins

**Fig. 1:**
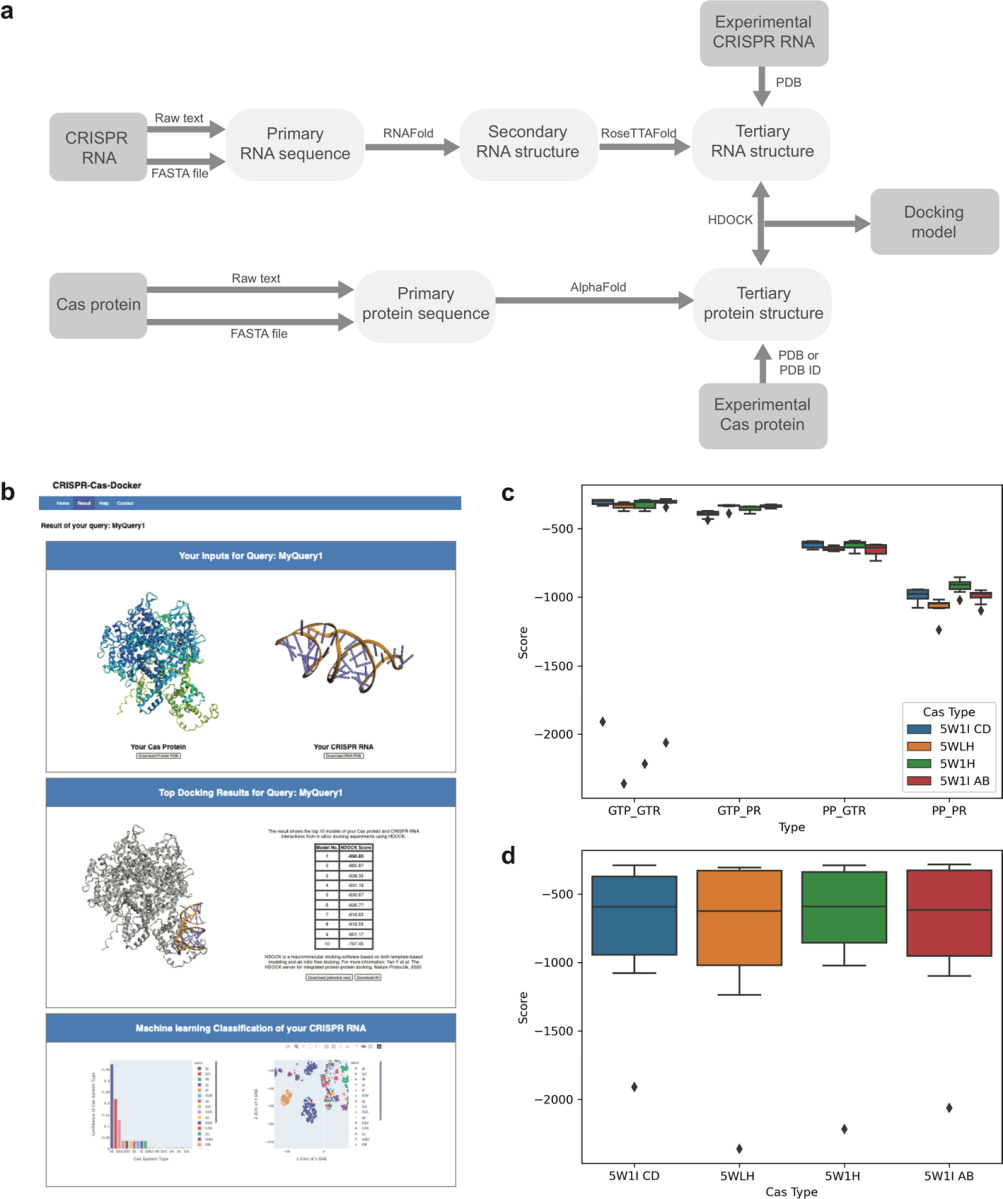
CRISPR-Cas-Docker. **a** Workflow used by CRISPR-Cas-Docker. **b** Results page generated by CRISPR-Cas-Docker, showing the downloadable PDB files of an AlphaFold-predicted Cas protein structure, a 3D-predicted crRNA structure, and the top-10 docking models. **c** Performance of CRISPR-Cas-Docker, using individual boxplots to show the docking scores obtained for different Cas13 proteins. **d** Performance of CRISPR-Cas-Docker, showing the distribution of docking scores obtained for different types of Cas proteins with GTP and PP combined. According to the HDOCK server, a lower docking score indicates a better docking model. (GTP: Ground Truth Cas Protein; GTR: Ground Truth crRNA; PP: Predicted Cas Protein (AlphaFold); PR: Predicted crRNA (RoseTTAFold))

In silico docking requires the availability of the 3D structures of biological macromolecules, which can be obtained through experimental techniques such as X-ray crystallography, NMR, and cryoelectron microscopy [12]. If experimentally determined structures are not available, these 3D structures can be estimated rapidly and accurately through (1) deep learning-based protein structure prediction programs such as AlphaFold [13, 14] and (2) a combination of 2D and 3D RNA structure prediction programs [15, 16]. Using the experimentally determined structures of Cas proteins, we verified that AlphaFold is able to achieve an adequate level of prediction accuracy for large effector proteins such as Cas13 (Additional file 1: Table S1). We used AlphaFold to model four Cas13 proteins with and without a template. The average (standard deviation) of the TM-score, defined as the maximum structural similarity between two proteins, normalized by the length of the longer protein, was 0.992 (0.001) and 0.817 (0.012), with and without a template, respectively. CRISPR-Cas-Docker has an integrated option to generate a 3D-predicted RNA structure and an AlphaFold-predicted protein structure for a crRNA sequence and a Cas protein sequence, respectively (Fig. 1a, b). The running time of CRISPR-Cas-Docker is affected by the length of a Cas protein sequence, as AlphaFold is the bottleneck of the computation process in the CRISPR-Cas-server (e.g. 2 h for 400 amino acids and 10 h for 1,400 amino acids).

### In silico docking of crRNAs and Cas proteins

In earlier work, we determined the best program to conduct in silico experiments between crRNAs and Cas proteins to be HDOCK [17], leading to the most accurate RNA–protein docking and binding affinity results using an experimentally validated dataset [11]. CRISPR-Cas-Docker uses the template-free docking approach of HDOCK to generate the top-10 docking models for a given crRNA-Cas protein pair, with the docking score of each model calculated by statistical mechanics-based energy scoring functions [18]. Previously, we verified that a docking score is a strong indicator of the binding affinity between crRNA-Cas protein complexes [11]. We compared the docking scores between all combinations of experimentally determined and computationally predicted crRNAs and Cas proteins (Additional file 1: Fig. S2). According to this performance study, AlphaFold-predicted proteins docked equally well or even better with the experimental crRNA and the 3D-predicted crRNA (Fig. 1c, d). From these results, we conclude that the effectiveness of docking is not affected by the use of predicted structures instead of experimental structures. The final step of CRISPR-Cas-Docker requires human expertise to identify the best in silico docking model from the generated top-10 docking models, using biological information such as the location of binding sites and the orientation of bound crRNA.

### Machine learning-based classification of crRNAs

CRISPR-Cas-Docker includes support for machine learning-based classification of an input crRNA sequence into its associated Cas system type [7–9]. This feature is a sequence-based prediction of the optimal Cas protein for a particular crRNA sequence, which is an alternative method to the structure-based prediction of optimal crRNA-Cas pairs. To learn the associations between CRISPR arrays and Cas systems, we first created a dataset of CRISPR arrays labeled with their co-localized Cas system type (Additional file 1: Fig. S3-S7). To that end, we extracted the CRISPR-Cas systems from the CRISPRCasdb [19] and labeled the CRISPR arrays co-localized within ± 10,000 base pairs with their corresponding Cas system (Additional file 1: Table S2). Next, we trained a K-Nearest Neighbors (KNN) algorithm on the curated dataset for supervised machine learning-based classification of crRNAs. Although KNN is one of the simplest classifiers in the area of machine learning, it has been used widely in the fields of gene and protein prediction, thanks to its interpretability, even when making use of complex data [20–23]. The classification analysis shows an overall prediction accuracy of 92.3%, confirming the ability of KNN to act as an accurate and efficient classifier of crRNAs into their associated Cas system type. Upon assessing the performance of individual classes, the major classes with over 1,000 data points demonstrated F1 scores above 0.89. For the classes with a lower number of data points, a substantial performance gap was observed (Additional file 1: Table S3, Figure S8).

## Conclusion

Designed for experimental biologists, CRISPR-Cas-Docker addresses the need to predict optimal crRNA-Cas protein pairs in silico before conducting expensive and time-consuming experiments. As metagenomic data become widely available, this bioinformatics tool enables performing a rapid preliminary study to disentangle the complex associations between multiple CRISPR arrays and Cas systems in prokaryotic genomes. Currently, CRISPR-Cas-Docker produces 3D-predicted structures of crRNAs and Cas proteins, top-10 docking models, and interactive graphs to visualize the machine learning-based classification of an input crRNA into its Cas system type. CRISPR-Cas-Docker is available as an easy-to-use and fully-integrated webserver with the aim of accelerating research in the CRISPR-Cas community by optimizing several computational tools and by providing a new evaluation method for CRISPR-Cas interactions. As future prospects, we aim at integrating AlphaFold-Multimer as a protein prediction program, making it possible to have Cas proteins with multi-unit effectors as an input to CRISPR-Cas-Docker.

## Availability and requirements

Project name: CRISPR-Cas-Docker. Project home page: http://www.crisprcasdocker.org/. Operating system(s): Platform independent. Programming language: Python 3.8.13. Other requirements: Web browser and internet access. License: GNU General Public License v3.0. Any restrictions to use by non-academics: None.

## Supplementary Information

**Additional file 1.** Supplemental Figures and Tables.

## Data Availability

The datasets generated and/or analysed during the current study are available in the CRISPR-Cas-Docker repository, https://github.com/hshimlab/CRISPR-Cas-Docker. 2D RNA structures and 3D RNA structures were predicted with ViennaRNA v.2.5.1 and RoseTTAFold v.2.0.0, respectively. In silico docking experiments were performed with HDOCK v.1.1.0. Protein structures were predicted with AlphaFold2, available under an open-source license at https://github.com/deepmind/alphafold. As protein structure similarity metrics, we used TM-align (https://zhanggroup.org/TM-align). 3-D structure visualizations were created with 3Dmol.js (https://3dmol.csb.pitt.edu/doc/tutorial-embeddable.html). For data analysis purposes, Python 3.8.13 (https://www.python.org), NumPy v.1.23.4 (https://github.com/numpy/numpy), Seaborn v.0.12.0 (https://github.com/mwaskom/seaborn), Matplotlib v.3.5.3 (https://github.com/matplotlib/matplotlib), and Pandas v.1.4.3 (https://github.com/pandas-dev/pandas) were used.

## Abbreviations

3D: Three-dimensional
2D: Two-dimensional
CRISPR: Clustered regularly interspaced short palindromic repeats
Cas: CRISPR-associated system
crRNA: CRISPR RNA
KNN: K-nearest neighbors
TM-score: Template modelling score

## References

[1] Makarova KS, Grishin NV, Shabalina SA, Wolf YI, Koonin EV (2006). A putative RNA-interference-based immune system in prokaryotes: computational analysis of the predicted enzymatic machinery, functional analogies with eukaryotic RNAi, and hypothetical mechanisms of action. Biol Direct.

[2] Mojica FJM, Díez-Villaseñor C, García-Martínez J, Soria E (2005). Intervening sequences of regularly spaced prokaryotic repeats derive from foreign genetic elements. J Mol Evol.

[3] Jinek M, Chylinski K, Fonfara I, Hauer M, Doudna JA, Charpentier E (2012). A programmable dual-RNA-guided DNA endonuclease in adaptive bacterial immunity. Science.

[4] Cong L, Ran FA, Cox D, Lin S, Barretto R, Habib N (2013). Multiplex genome engineering using CRISPR/Cas systems. Science.

[5] Bikard D, Euler CW, Jiang W, Nussenzweig PM, Goldberg GW, Duportet X (2014). Exploiting CRISPR-Cas nucleases to produce sequence-specific antimicrobials. Nat Biotechnol.

[6] Citorik RJ, Mimee M, Lu TK (2014). Sequence-specific antimicrobials using efficiently delivered RNA-guided nucleases. Nat Biotechnol.

[7] Koonin EV, Makarova KS, Wolf YI (2017). Evolutionary genomics of defense systems in archaea and bacteria. Annu Rev Microbiol.

[8] Makarova KS, Wolf YI, Alkhnbashi OS, Costa F, Shah SA, Saunders SJ (2015). An updated evolutionary classification of CRISPR–Cas systems. Nat Rev Microbiol.

[9] Makarova KS, Wolf YI, Iranzo J, Shmakov SA, Alkhnbashi OS, Brouns SJJ (2019). Evolutionary classification of CRISPR–Cas systems: a burst of class 2 and derived variants. Nat Rev Microbiol.

[10] Shim H (2022). Investigating the genomic background of CRISPR-Cas genomes for CRISPR-based antimicrobials. Evol Bioinform Online.

[11] Park H-M, Park Y, Berani U, Bang E, Vankerschaver J, Van Messem A (2022). In silico optimization of RNA-protein interactions for CRISPR-Cas13-based antimicrobials. Biol Direct.

[12] Berman HM, Westbrook J, Feng Z, Gilliland G, Bhat TN, Weissig H (2000). The protein data bank. Nucleic Acids Res.

[13] Jumper J, Evans R, Pritzel A, Green T, Figurnov M, Ronneberger O (2021). Highly accurate protein structure prediction with AlphaFold. Nature.

[14] Park H-M, Park Y, Vankerschaver J, Van Messem A, De Neve W, Shim H (2022). Rethinking protein drug design with highly accurate structure prediction of anti-CRISPR proteins. Pharmaceuticals.

[15] Antczak M, Popenda M, Zok T, Sarzynska J, Ratajczak T, Tomczyk K (2017). New functionality of RNAComposer: application to shape the axis of miR160 precursor structure. Acta Biochim Pol.

[16] Cheng CY, Chou F-C, Das R (2015). Modeling complex RNA tertiary folds with Rosetta. Methods Enzymol.

[17] Yan Y, Tao H, He J, Huang S-Y (2020). The HDOCK server for integrated protein-protein docking. Nat Protoc.

[18] Huang S-Y, Zou X (2010). MDockPP: a hierarchical approach for protein-protein docking and its application to CAPRI rounds 15–19. Proteins.

[19] Couvin D, Bernheim A, Toffano-Nioche C, Touchon M, Michalik J, Néron B (2018). CRISPRCasFinder, an update of CRISRFinder, includes a portable version, enhanced performance and integrates search for Cas proteins. Nucleic Acids Res.

[20] Ayyad SM, Saleh AI, Labib LM (2019). Gene expression cancer classification using modified K-Nearest Neighbors technique. Biosystems.

[21] Arian R, Hariri A, Mehridehnavi A, Fassihi A, Ghasemi F (2020). Protein kinase inhibitors’ classification using K-Nearest neighbor algorithm. Comput Biol Chem.

[22] Ning Q, Ma Z, Zhao X (2019). dForml(KNN)-PseAAC: Detecting formylation sites from protein sequences using K-nearest neighbor algorithm via Chou’s 5-step rule and pseudo components. J Theor Biol.

[23] Ding Y, Yang C, Tang J, Guo F (2022). Identification of protein-nucleotide binding residues via graph regularized k-local hyperplane distance nearest neighbor model. Appl Intell.

